# Editorial: Zoonotic emerging viral infectious diseases

**DOI:** 10.3389/fvets.2023.1194324

**Published:** 2023-04-11

**Authors:** Ji-Ming Chen, Yu-Fei Ji, Zhao-Jun Duan, Bin Wei

**Affiliations:** ^1^School of Life Science and Engineering, Foshan University, Foshan, China; ^2^National Institute for Viral Diseases Control and Prevention, Chinese Center for Disease Control and Prevention, Beijing, China; ^3^School of Life Sciences, Shanghai University, Shanghai, China

**Keywords:** virus, zoonosis, emerging infectious disease, influenza, monkeypox, rabies

Most emerging virus infectious diseases in humans, such as H5N1 and H7N9 subtypes of avian influenza (1), Middle East respiratory syndrome (MERS) (2), Ebola virus disease (EVD) (3), COVID-19 (4), monkeypox (Mpox) (5), and Marburg virus Disease (6) are zoonotic in nature. These zoonotic emerging viral infectious diseases can have devastating effects on livestock and poultry production, resulting in significant economic losses and posing serious threats to public health (1, 7).

In order to improve the prevention and control of zoonotic emerging viral infectious diseases, further research is necessary to identify and characterize unknown viruses in both domestic and wild animals. It is important to monitor the diversity, distribution, evolution, and potential risks of various viruses that have the potential to cause zoonosis. Additionally, it is crucial to clarify the pathogenesis of these viruses and develop effective strategies and measures for detecting, surveying, preventing, controlling, and eliminating zoonotic emerging viral infectious diseases. These studies are essential for risk analysis and policy-making in animal protection and public health, and are therefore significant areas of research in veterinary and medical sciences.

Recently, Frontiers in Veterinary Science published six articles on the topic of *Zoonotic Emerging Viral Infectious Diseases*. This Editorial is to summarize the findings and significance of these six papers.

The first article provides a summary of the epidemiology of monkeypox (Mpox), which has spread to over 100 countries since May 2022. Through a literature review, this article highlights the risks associated with this outbreak and proposes eight strategies for its elimination, primarily based on the summarized epidemiology (Chen et al.). It suggests that strengthened control measures have helped to shift the outbreak from a rapidly increasing trend to a declining one. However, some American countries are still experiencing sustained community transmission of Mpox, and it is crucial to enhance control measures in these regions (8). Without such measures, the virus may continue to circulate in humans and adapt to humans through genomic mutation, potentially sparking a new pandemic with disastrous consequences.

The second article presents evidence for the identification and genomic characterization of a new porcine parvovirus (PPV) in China, tentatively named PPV8 (Guo et al.). This virus can represent a new species in the genus of *Protoparvovirus*, which currently includes three species of carnivore parvoviruses, four species of primate parvoviruses, three species of rodent parvoviruses, two species of ungulate parvoviruses (including porcine or bovine parvoviruses), and one species of bat parvovirus (9). The PPV8 was found in the lung samples of sick pigs collected in China between 1990 and 2021. However, it is unclear whether this novel porcine parvovirus has the potential to infect humans, and therefore its zoonotic potential should be clarified in the future.

The third article reports a novel approach for the on-site detection of bovine coronavirus (BCoV) infection in suspected farms that is rapid, reliable, and simple (Ji et al.). BCoV is a major cause of infectious disease in cattle worldwide and can infect humans and multiple other species of mammals. The authors developed a multienzyme isothermal rapid amplification (MIRA) and lateral flow dipstick (LFD) combination assay, which targets a highly conserved region of the viral nucleocapsid (N) gene for BCoV detection. This MIRA-LFD assay is superior to classical real-time PCR assays as it does not require sophisticated instruments and the results can be visually observed. Furthermore, this method has the potential to be used for the on-site detection of other zoonotic viruses.

The fourth article describes the varying perspectives among veterinarians in the USA on swine influenza virus (SwIV) infections in pigs (Moraes et al.). The study also highlights the veterinarians' belief that SwIV is becoming a more significant health issue in swine. SwIV infection was the second most commonly confirmed disease in respiratory porcine tissue cases in some regions in the USA between 2010 and 2019 (10). SwIV can also infect humans, and human influenza virus can infect pigs, posing a threat to both species. The authors recommended strengthening or optimizing influenza vaccination in both swine and humans, as well as improving biosecurity measures. However, we believe that vaccinating pigs with low-efficacy vaccines against SwIV may accelerate the antigenic mutation and diversification of the virus, making the disease more complex (11). In countries like China, where SwIV vaccination is not widespread, SwIV infections in pigs may be less devastating (personal communication).

The fifth article describes the emergence of a human-porcine reassortment porcine rotavirus A strain in China (Luo et al.). Group A rotaviruses of the family *Reoviridae* is one of the important intestinal pathogens causing diarrhea in piglets and humans. A human-porcine reassortment rotavirus was identified from outbreak of diarrhea in suckling piglets with 60.00% (324/540) morbidity and 20.99% (68/324) mortality in Guangdong Province of China in 2022. The virus strain likely emerged as the result of genetic reassortment between porcine and human rotaviruses, suggesting that the strain has a potential risk of cross-species transmission.

The six article focuses on rabies virus in white-nosed coatis (*Nasua narica*) in Mexico (Puebla-Rodríguez et al.). Although Mexico has successfully eliminated human rabies transmitted by dogs, the country still faces the challenge of wildlife transmitting the virus to humans and domestic animals. The study reports 13 cases of rabies in white-nosed coatis in Mexico between 1993 and 2022. The rabies virus strains from nine of the samples were antigenically and genetically characterized. Coatis have not been previously considered important vectors of rabies virus, but the significance of rabies virus infections in coatis should be carefully monitored in order to prevent human infections transmitted by this species.

In summary, the six articles mentioned above make significant contributions to risk analysis and the selection of control strategies for zoonotic emerging viral infectious diseases. They also aid in identifying new viruses with zoonotic potential, developing new techniques for on-site detection of zoonotic viruses, and enhancing surveillance of zoonotic emerging viral infectious diseases. These articles underscore the importance and necessity of conducting studies on various aspects related to zoonotic emerging viral infectious diseases.

## Author contributions

J-MC and Y-FJ wrote this editorial. Z-JD and BW revised it. All authors approved the submitted version.
